# From design to decision-making: emergency manager participation in the development of coastal storm decision support tools

**DOI:** 10.1007/s44367-025-00022-2

**Published:** 2025-11-13

**Authors:** Noah Hallisey, Austin Becker, Peter Stempel, Olivia Krum

**Affiliations:** 1 Department of Marine Affairs, University of Rhode Island, Kingston, RI 02881 USA; 2 Department of Landscape Architecture, The Pennsylvania State University, State College, PA USA

**Keywords:** Emergency management, Decision support tool, Coastal storms, Co-development

## Abstract

**Introduction:**

Computer-based decision support tools (DSTs) are indispensable to emergency managers (EMs). Despite their proliferation in recent years, there is a persistent disconnect between the tools developed and their use. Developing tools with end-users has been recommended to ensure they have practical relevance and achieve widespread adoption and utilization. While existing work has primarily focused on DST development, end-user engagement in DST development and implementation has received less attention. In this paper, we review the existing literature on coastal storm DST development to determine whether and how researchers are engaging EMs in the development of DSTs and their reporting on the outcomes of these efforts.

**Methods:**

We conducted a literature review of coastal storm DST development, looking for mentions of EM engagement. Our search identified 286 articles, and the review was conducted on 65 of the articles that mentioned a DST, of which six explicitly mentioned engaging EMs in development.

**Results:**

Of the 286 articles we identified, only six articles explicitly report engaging EMs in tool development, suggesting low levels of engagement. We found limited reporting on the outcomes of these efforts. We identified four approaches from the six articles reviewed: (1) conduct a needs identify emergency managers’ needs to guide the development; (2) from a collaborative team to engage emergency managers in tool development; (3) host workshops and training exercises with emergency managers using the tool to solicit feedback; and (4) use an iterative design approach to incorporate EM feedback into the tool.

**Conclusion:**

Findings of our review reveal low levels of EM engagement reported in existing literature, which may reflect a disconnect between the tools being developed and their use by EMs. We also found limited reporting on the outcomes of these efforts, suggesting that most DSTs have yet to achieve mainstream use. Recommendations for research and practice include (1) increased reporting on the engagement of EM in DST development; (2) an assessment of the barriers related to DST development and implementation for both tool developers and EMs; (3) longitudinal studies or case studies of DST development and implementation; and (4) the creation of a standardized approach or set of best practices for DST development and implementation to increase their success.

## Introduction

The intensification of coastal storms (e.g., nor’easters, hurricanes, cyclones, and typhoons) threatens coastal communities worldwide (Balaguru et al., 2022; Young & Hsiang, 2024). In response to the growing threat, researchers advance computer-based decision support tools (DSTs) that disseminate critical information regarding storm hazards (wind, wave, storm surge, and flooding) and their impacts to inform emergency manager (EM) decision-making (Adams et al., 2024; Rucker et al., 2021). Researchers claim that these tools enhance EM decision-making capacity; however, despite their proliferation in recent years, researchers continue to experience challenges developing DSTs that meet the needs of EM and achieve widespread adoption and use (Little et al., 2015a; Newman et al., 2017; Pearman & Cravens, 2022; Poch et al., 2017). These challenges are attributed to a lack of EM engagement in DST development, resulting in tools that lack practical application because EM needs are not adequately reflected in the tool (Little et al., 2015b), as well as issues related to the adoption and implementation of DSTs (McIntosh et al., 2011). Recognizing these challenges, researchers promote adopting a collaborative approach to tool development through co-development to overcome the challenges of DST development and implementation, in which researchers and EM work collectively to conceptualize and develop tools that better meet EM needs and are more likely to serve a practical function and therefore be adopted and used (Fogli & Guida, 2013; Little et al., 2015a; Pearman & Cravens, 2022). Despite such recommendations to overcome the persistent failure of DST to meet EM needs and achieve widespread use, there is little research investigating approaches for engaging EM in their development or any standardized guidance or recommendations for improving development and implementation, and the existing research evaluating DST development and adoption has reported minimal end-user engagement and success in achieving widespread use (Newman et al., 2017; Walling & Vaneeckhaute, 2020). Increasingly deadly and costly storm events mean that EM need access to these tools to ensure they make informed decisions that reduce damage and save lives now more than ever. Yet, it remains unclear whether researchers are using approaches that engage EM in DST development for the management of coastal storms, and if such, efforts are successful in overcoming the challenges related to development and implementation. In this paper, we review existing literature to identify if and how researchers report on engaging EM in the development of DSTs for coastal storm management, highlighting the approaches being employed and reporting on reporting on their outcomes. This review addresses the following research questions (RQ):

- RQ1: How does the peer-reviewed literature describe researcher engagement efforts with EM in the development of DSTs?
- RQ2: What approaches are researchers using to engage EM in the development of DSTs?

This paper examines existing literature to assess the extent and ways researchers engage EM in developing new DSTs for coastal storm management. It maps out the current state of knowledge regarding the co-development of coastal storm DSTs, identifying existing and prevalent theories, as well as existing gaps and makes recommendations on areas for future research. We begin with an overview of the issue, then present the methodology used to conduct the literature review, followed by an interpretation of our findings. We then present the four approaches used to engage EM from the articles we reviewed, which future efforts could adopt and expand upon to increase EM engagement in DST development. We conclude with recommendations for future research.

## Background

There is broad agreement that computer-based DSTs play a critical role in the management of a multitude of disasters, including coastal storms (Little et al., 2015a; Newman et al., 2017; Stoltz et al., 2023). DSTs consist of a suite of natural hazards-related information (e.g., storm models), advanced analytical capabilities, and a graphical user interface (GUI) that disseminates relevant and timely information to decision-makers, such as EM (Lindell & Prater, 2007; Wong-Parodi et al., 2020). In recent years, DSTs have become an increasingly popular approach to support decision-making related to the management of coastal storm hazards, as well as other applications for natural hazards and environmental management (Barzehkar et al., 2021; Newman et al., 2017; Walling & Vaneeckhaute, 2020; Yaqoob et al., 2014). For example, the United States Federal Emergency Management Agency (FEMA) developed the HURRicane EVACuation (HURREVAC) tool. EM use HURREVAC to inform hurricane evacuation decision-making, as well as assess storm risk to communities and infrastructure using outputs generated from high-resolution prediction models (Glahn et al., 2009). Other examples of DSTs that support EM with the coastal storm management include the Hurricane Evacuation Management Decision Support System (EMDSS), a hurricane evacuation DST developed by Lindell et al. (2006), the MUNICIPAL TOOL, a tool simulating storm impacts to critical infrastructure developed Little et al. (2015b), and the Coastal Hazards Analysis Modeling and Prediction (CHAMP) system, a hazard and impact prediction systems designed to support EM with storm preparedness and response decision-making created by Adams et al. (2024).

EM play a critical role in ensuring the safety and well-being of the public during coastal storms, and storms are making their job more challenging (Hoekstra & Montz, 2017; Little et al., 2015a). Escalating coastal storms are causing more deaths and damage now and into the coming years. For example, the 2024 Atlantic Hurricane season was one of the deadliest on record, with over 150 people killed and a damage cost surpassing $190 billion (Allen, 2024). Because of patterns of intensifying storms in recent years, researchers are investing significant resources and time into advancing the informational products and tools available to support emergency manager decision-making, including the development of predictive storm models and associated DSTs that predict coastal flooding and other storm-related hazards (Fleming et al., 2008; Rucker et al., 2021). Such models are being integrated into DSTs that support real-time planning and response, such as CHAMP (Adams et al., 2024). EM can use the timely and localized information provided by these tools to inform decisions that reduces a storm’s physical, social, and economic impacts (Burston et al., 2015; Chen et al., 2011; Davidson et al., 2020).

DSTs have become increasingly popular because they serve as boundary object bringing together scientists (e.g., researchers) and non-scientists (e.g., EM) to co-produce actionable information and tools that can benefit decision-makers in varying contexts (Pearman & Cravens, 2022; Stoltz et al., 2023). Yet, despite their growing prevalence in recent years, persistent challenges related to development and implementation prevent their widespread adoption and use (McIntosh et al., 2011; Newman et al., 2017; Pearman & Cravens, 2022; Poch et al., 2017; Stoltz et al., 2023; Walling & Vaneeckhaute, 2020). The challenges of effectively developing and implementing DSTs are often attributed to a lack of engagement of end-users in their development (Little et al., 2015b; McIntosh et al., 2011; Newman et al., 2017), as well as difficulties related to adoption and implementation, such as determining ownership and maintenance once DSTs are operational (McIntosh et al., 2011; Poch et al., 2017). Researchers often possess a keen and relevant understanding of storm modeling for developing practical DSTs. However, they lack an understanding of the operational and technical requirements of EM, including the kinds of decisions that will be made using the tool (Little et al., 2015b; Walling & Vaneeckhaute, 2020). EM require actionable information to inform decision-making, which researchers often struggle to deliver through the tools they create (Merz et al., 2020). This mismatch in the cross-scale communication patterns between expert and non-expert communities affects the resource distribution and ultimately hinders their development, adoption, and real-time use (Jack, 2006; Little et al., 2015a; Olman & DeVasto, 2021). These challenges are problematic because researchers invest considerable time and resources to develop and implement these tools, yet struggle to get them into the hands of decision-makers, and therefore, valuable time and resources are wasted (Walling & Vaneeckhaute, 2020).

Recognizing the low success rates of efforts to develop and implement DSTs, researchers have recommended a shift in approach to DST development that engages EM from conception to implementation (Fogli & Guida, 2013; Little et al., 2015a). Researchers posit that such an approach will lead to increased use of DSTs because they will better meet the needs of emergency managers, and through engaging EM, they will get the buy-in needed to support transitioning their tools into operational products that decision-makers use. However, previous research has noted persistent challenges related to end-user engagement caused by issues related to a lack of engagement strategies and funding to support engagement (Pearman & Cravens, 2022; Stoltz et al., 2023; Walling & Vaneeckhaute, 2020). And when EM are excluded from tool development, they are often more hesitant to adopt them (Turanitza, 2018). As a result, efforts to develop and implement DSTs are more likely to fail. For example, findings from an evaluation of DSTs for the management of natural hazards observed low rates of EM engagement in their development, and no reporting on their successful implementation (Newman et al., 2017).

Researchers often claim that these tools can benefit decision-makers; however, such claims are not substantiated due to the challenges associated with development and implementation, which often result in tools being underutilized or unused. Despite recognition of these, there is an overall lack of work related to the engagement of EM in DSTs, including an assessment of EM engagement in their development rates, standardized approaches for engaging end-users in development, and a lack of reporting on the success of these efforts (Newman et al., 2017; Walling & Vaneeckhaute, 2020). In this paper, we review existing literature on DSTs for the management of coastal storms to evaluate the extent to which EM are being engaged, the approaches researchers are using to engage EM, and report on the outcomes of these efforts to address these gaps.

## Methods

We conducted a review to evaluate whether and how researchers described their engagement with EM in developing coastal storm DSTs in existing literature. Here, *tools* are defined as software products designed to aid and inform EM about coastal hazards in advance of and/or during an event. *Engagement* is defined as approaches used by researchers that include EM in the conceptualization, design, development, or implementation of a DST through approaches such as soliciting feedback, conducting workshops or focus groups, and other such processes for incorporating end-user needs directly into tool development. For this study, we define coastal storm DSTs as computer-based tools providing EM with information regarding storm hazards intended to support their decision-making. We focus on coastal storms DSTs as previous research has recommended the engagement of EM as a normative approach to their development (Little et al., 2015a). Additionally, they pose the greatest threat to coastal communities throughout the world. For our review, we classified coastal storms as cyclones, hurricanes, and typhoons, using the geographical naming convention based on where they form. Our research builds from and advances findings from a recent review of DSTs for natural hazards management conducted by Newman et al. (2017), which reviewed articles published between 1995 and 2015, with our review specifically focusing on DSTs for the management of coastal storms. Our review covers literature from 2016 to 2024. To ensure our review was thorough, we searched two databases: Scopus and Web of Science. The primary target of our literature review is a survey of white literature (i.e., published research). Because our focus was on DSTs developed by researchers, we focused on reviewed peer-reviewed journal articles reporting on the development of DSTs, specifically focusing on how researchers are reporting on EM engagement in DST development. We considered surveying grey literature to incorporate government reports; however, we refrained from including grey literature as our primary focus was on existing academic literature where researchers tend to share their work. To identify articles, we searched for the following terms in combination with the title, abstract, and article keywords (Table 1):

**Table 1:** Search terms used for review and # articles returned for each search in Scopus and Web of Science and total number once duplicates were removed

Search term phrases	Scopus	Web of Science	Total w/duplicates removed
Cyclone & Decision & Support & Tool	21	16	23
Cyclone & Decision & Support & Technology	17	12	18
Cyclone & Decision & Support & System	84	43	95
Cyclone & Decision & Support & Tool & Emergency & Management	2	2	2
Cyclone & Decision & Support & Technology & Emergency & Management	3	2	4
Cyclone & Decision & Support & System & Emergency & Management	5	2	5
Hurricane & Decision & Support & Tool	42	26	46
Hurricane & Decision & Support & Technology	15	10	21
Hurricane & Decision & Support & System	141	61	153
Hurricane & Decision & Support & Tool & Emergency & Management	2	2	7
Hurricane & Decision & Support & Technology & Emergency & Management	3	0	3
Hurricane & Decision & Support & System & Emergency & Management	20	5	21
Typhoon & Decision & Support & Tool	18	5	82
Typhoon & Decision & Support & Technology	14	12	18
Typhoon & Decision & Support & System	75	38	82
Typhoon & Decision & Support & Tool & Emergency & Management	0	0	0
Typhoon & Decision & Support & Technology & Emergency & Management	4	2	6
Typhoon & Decision & Support & System & Emergency & Management	12	5	14
Total	483	243	286

We used the AND Boolean operator between each term as it returned more results for each search term as compared to using loose phrases (e.g., “decision support tool”). In addition, it allowed for any combination of search terms without them needing to be next to each other. We conducted our searches from 01/16/2025 to 01/19/2025. We then combined the results from both database searches and removed duplicate articles.

**Fig. 1:**
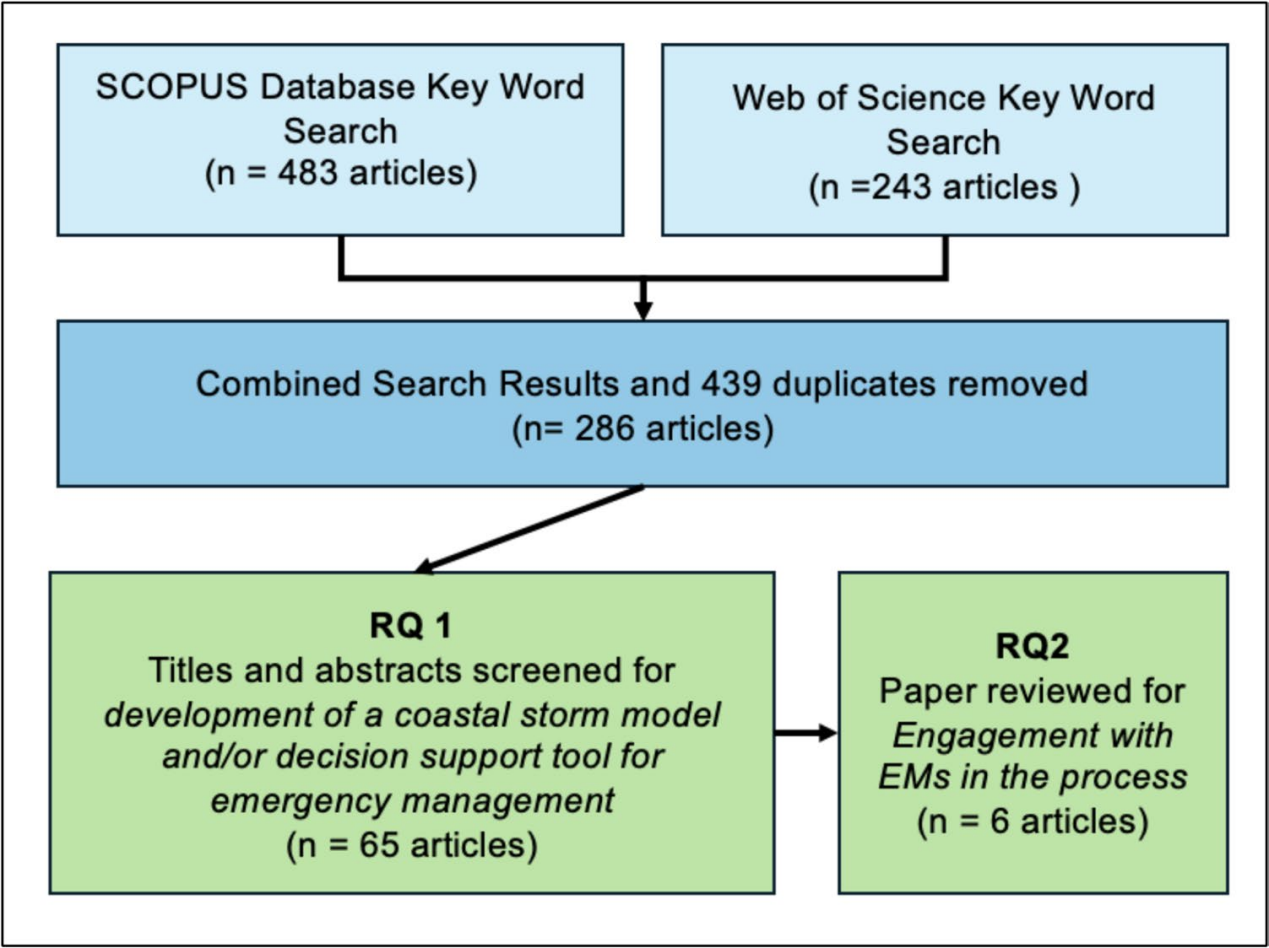
Flow chart for selection of reviewed articles

After removing 439 duplicate articles, our initial sample totaled 286 articles. Each article abstract was scanned, and we removed articles that did not discuss the development of a coastal storm model and/or DST for emergency management*.* This left 65 articles to be reviewed. Each of the 65 articles was reviewed in depth for mentions of *engagement with* EM, as well as claims for how the tool benefited emergency management. After this stage, our sample consisted of six peer-reviewed articles that met the ultimate two criteria of interest for this review (Fig. 1):

- *Development of a coastal storm model and/or DST for emergency management*
- *Engagement with EM in the process*

The next section provides the results of our search and a discussion of our findings.

## Results

This section presents results of the review and discussion of the two research questions, taking each in turn. It begins with an analysis of the 65 articles that discussed the development of coastal storm models and/or DSTs for emergency managers to address the first questions. The section question is answered through the review of six articles that go into detail about how EM were engaged.

1. *RQ1 How does the peer-reviewed literature describe researcher engagement efforts with EM in the development of DSTs?*

We reviewed 65 of 286 articles that discussed the *development of a coastal storm model and/or DSTs for emergency management* to evaluate the extent to which EM were engaged in tool development. Twenty-nine of these articles framed their discussion as developing a tool that would ultimately be made applicable to specific EM end users.^1^ Such claims of their utility included supporting real-time decision-making (e.g., evacuation decision support), conducting risk assessments, informing emergency procedures, and strengthening storm preparedness and impact mitigation. We have included several examples of their merit in Table 2.

**Table 2:** Example language from the 65 articles that discussed the potential benefits of their tools for emergency management

Citation	Sample framing language describing the benefits of tools to end users
Xie et al. (2021)	*“The visualization system (i.e., SOSWWS) established based on this method, which contains forecast probabilities for exceedance of relevant thresholds, could provide swift response to the emergency management need and deliver reliable decision-making support.”*
Kushabaha et al. (2024)	*“Through advanced remote sensing, GIS analysis, and the development of a relational geodatabase and Web-GIS platform, we have produced a tool capable of providing critical information to decision-makers and promoting collaboration between stakeholders.”*
Chen and Liu (2016)	*“The inundation hazard maps developed in this study could be a useful tool for planning the evacuation of residents living in low-lying areas and the decision-making of flood risk management on the coasts and floodplains.”*
Watson et al. (2024)	*“This modeling approach has potential to inform decision makers about the overall severity of storm impacts, as well as which regions are more likely to be more affected by persistent power outages. This information could allow emergency managers to prepare more effectively and reduce the felt impacts of these storms.”*
Xianwu et al. (2020)	*“The proposed method could be easily adopted in various coastal counties and serves as an effective tool for the decision making in disaster risk reduction practices.”*
Liu et al. (2023)	*“This study offers a valuable tool for decision-makers to develop scientific strategies in the risk management of TC disaster”*

Of the twenty-nine articles, only six mentioned the specific identifying end-users, which were included in our review. We also evaluated the claims made in the articles regarding how tools could support EM, and if they were being substantiated through evidence of their adoption for real-time use. In other words, was the tool developed for EM actually being used? Of the six articles, only one reported successfully implementing their tool, and another reported being in the process of implementing it. Another article reported that their tool was used for training exercises. Two additional articles described an anticipated future engagement with EM end-users but were not included with the six that described actual EM engagement (Table 3).

**Table 3:** Claims regarding potential application compared to level of engagement as a percentage of articles

Claimed level of engagement with EM	Articles	Percent of total
Made claims regarding potential real-time use without engaging EM	29	45
Described an anticipated future engagement	2	3
Actually engaged articles	6	9

- *RQ2—What approaches are researchers using to engage EM when developing DSTs?*

Our search across 286 articles and a deeper review to search for details of EM engagement in the 65 articles returned only six that discussed engaging EM in the developmental process (Table 2). In addition to these six articles, one mentioned planning to partner with EM in the future to develop a DST, noting that they realized that the model they developed likely was insufficient in meeting the needs of EM, and developing a DST was necessary (Watson et al., 2024). Another article mentioned disseminating storm model outputs to EM for decision support, but made no mention of their engagement (Rucker et al., 2021). These two articles were not included in the detailed review.

Of the six articles reviewed, each presented a unique project in which a DST was an outcome. Five of the projects were in the USA, and one was in China. The tools identified through the review were designed to serve varying decision support purposes. Two were DSTs: one supporting evacuation planning and the other disaster preparedness and impact assessment. Two were simulation-based tools for hurricane preparedness. Another was a graph-powered visualization application for situational awareness, and the final was a hazard and impact prediction system for hurricane preparedness and response. All tools included a web-based graphic user interface (GUI) that was made available to EM and other decision-makers. GUI were developed using platforms including Spring Boot (Java Platform), GeoNode, Deck GL, Visual Basic, and ESRI Dashboards.

As part of this review, we synthesized and reported on engagement approaches mentioned, which include the following four approaches: (1) conduct a needs assessment to identify emergency managers needs to guide development of DST; (2) form collaborative team to engage emergency managers in tool development; (3) host workshops and training exercises with emergency managers using the tool to solicit feedback; and (4) use an iterative design approach to incorporate emergency manager feedback into the tool (Table 4).

**Table 4:** Articles that mentioned engagement with emergency managers in the development of a DST and the approach(es) they used

Article title	Authors	Year published	Tool	Approach mentioned
Contraflow evacuation e-planning for I-65 in Alabama	Moynihan & Froncesca	2016	Evacuation e-planning system for I-65 in Alabama	1
The Hurricane Decision Simulator: A Tool for Marine Forces in New Orleans to Practice Operations Management in Advance of a Hurricane: Finalists- 2017 M&SOM Practice Based Research Competition	Regneir & MacKenzie	2019	Hurricane Decision Simulator	1,3
Geospatial environments for hurricane risk assessment: applications to situational awareness and resilience planning in New Jersey	Kijewski-Correa et al.	2020	NJCoast and the Storm Hazard Projection (NHP) Tool	1,3,4
Facilitating Typhoon Flood Disaster-Ready Information Delivery Using SDI Services Approach – A Case Study in Hainan	Hu et al.	2022	Remote Sensing Disaster Decision Support System (RDDSS)	1
GeoGraphVis: A Knowledge Graph and Geovisualization Empowered Cyberinfrastructure to Support Disaster Response and Humanitarian Aid	Li et al.	2023	GeographVis	3,4
Ocean state rising: Storm simulation and vulnerability mapping to predict hurricane impacts for Rhode Island’s critical infrastructure	Adams et al.	2024	RI-CHAMP	1,2,3,4
Code definitions 1: Conduct needs assessment to identify EM needs to guide development of DST 2: Form collaborative team to engage emergency managers in tool development 3: Host workshops and training exercises with emergency managers using the tool to solicit feedback 4: Use an iterative design approach to incorporate emergency managers feedback into the tool				

In the following section, we provide details regarding the reporting on the engagement approaches in the articles we reviewed. We then discuss these findings and how they address the RQs in more detail.

### Approach 1, conduct needs assessment to identify EM needs to guide development of DST

Five of the six articles described a needs assessment process as part of the development of the tool. DSTs must meet EM needs to provide real-time assistance. Otherwise, they may go unused. Researchers recognize the importance of incorporating EM needs into these tools to ensure they best support decision-making (Burston et al., 2015; Morss et al., 2022; Munroe et al., 2018). Yet, they often struggle to develop tools that address EM needs because they are not adequately understood. An approach to better understanding the needs of EM that was identified through our review was to conduct a needs assessment (Adams et al., 2024; Kijewski-Correa et al., 2020). Needs assessments identify stakeholder requirements that inform the creation of outputs or outcomes specifically addressing these needs and result in more appropriate interventions (Kaufman & English, 1979). Approaches to conducting needs assessment can include surveys, interviews, and focus groups with stakeholders to identify needs (Royse et al., 2009). Needs assessments can be used to identify potential challenges and opportunities for addressing the need, prioritize action, and leverage resources towards effective interventions (Gupta, 2011). In the development of a tool to support emergency managers in NJ, USA, called NJCoast, researchers conducted a needs assessment using generative sessions to identify the data needs of emergency managers, develop an understanding of the operational workflow and procedures of emergency managers, and determine the technical capacity of its adopters which guided the design of NJCoast (Kijewski-Correa et al., 2020). In the development of a DST for emergency managers in RI, USA, called the Rhode Island Coastal Hazards, Analysis, Modeling, and Prediction (RI-CHAMP) system, researchers worked with emergency managers to identify their specific data needs regarding storm impacts to critical infrastructure and reflected them in RI-CHAMP (Adams et al., 2024). This approach is referred to as “decision-driven data system,” in which end-user data needs used to inform the design of systems that better support decision-making (Cantor et al., 2021). A needs assessment conducted early in the research process can identify the specific end-users of the tool and shape its design and functionality (Kijewski-Correa et al., 2020. Needs assessment used in this context has been recommended previously (Little et al., 2015a), and is further supported by our findings.

### Approach 2, form collaborative team to engage emergency managers in tool development

One article described the formation of a collaborative team (Adams et al., 2024). EM have noted that they do not feel researchers sufficiently engage them when developing new tools, and because they are not involved, they are less likely to adopt the tool (Turanitza, 2018). Therefore, researchers must use approaches that catalyze EM engagement in the initial stages of development. Our review identified several ways in which researchers could accomplish such engagement. In the development of RI-CHAMP, researchers formed a steering committee, connecting federal, state, and local EM with the research team (Adams et al., 2024). A steering committee consists of stakeholders that guide a project from its onset, steering it towards achieving its intended goals and facilitating implementation of project outcomes upon completion (Lechler & Cohen, 2009). Steering committees facilitate interaction between researchers and decision-makers, ushering a research process that is pragmatic and results in research outputs easily translatable into useful tools for decision-makers (Keita et al., 2017). Researchers spearheading RI-CHAMP noted that the steering committee played a critical role in guiding the entirety of the project, from early conception to the delivery of a dashboard tailored to EM needs (Adams et al., 2024).

### Approach 3, host workshops and training exercises with emergency managers using the tool to solicit feedback

Four articles described the use of training exercises in the development of the tool. A lack of training and resources in the use of new tools has been cited as a barrier to their adoption, especially among emergency management agencies that infrequently utilize DSTs (Little et al., 2015a). Coastal storms are infrequent. Therefore, EM may have limited opportunities to put a new tool to the test. Because of their limited use, EM can be unfamiliar with these tools. This can be problematic during real-time events; EM must make timely decisions in high-stress situations and often lack time or bandwidth to learn new tools on the fly. Therefore, training and workshops may be required to facilitate the adoption of new tools (Turoff et al., 2004). Workshops and training exercises were identified as a useful approach for training EM on the new tool, as well as providing opportunities to give feedback on the tool based on experiences using it during simulated events (Adams et al., 2024; Kijewski-Correa et al., 2020; Regnier & MacKenzie, 2019). Researchers developing CHAMP hosted a workshop with local and state emergency managers, testing the tool in three hypothetical storm scenarios. Researchers noted that these exercises allowed emergency managers to interact with the tool and evaluate its utility for supporting decision-making during a real-time event, as well as collect feedback that could be incorporated (Adams et al., 2024). Researchers developing the Hurricane Decision Simulator utilized a similar approach, in which they led two group hurricane preparation exercises with over 40 participants each year, in addition to a yearly tabletop exercise (Regnier & MacKenzie, 2019). These exercises supported further development and refinement of the Hurricane Decision Simulator while simultaneously allowing EM to enact preparedness and response plans, gaining practical experience and refining existing plans (Regnier & MacKenzie, 2019).

### Approach 4, use an iterative design approach to incorporate emergency managers feedback into the tool

Three articles cited the incorporation of intuitive design approaches to ensure EM feedback was incorporated into tool development. Fine-tuning a tool to the needs of EM requires an iterative approach through cycles of testing and evaluation (Little et al., 2015a). Based on our review, researchers adopted several approaches to facilitate this process. The first was an Agile development process (Kijewski-Correa et al., 2020; Li et al., 2023). The Agile development process is an iterative workflow derived from the Manifesto for Software Development, by which software developers test their products with end-users, and further modifications are made based on user feedback and evaluation, resulting in a final product with greater value to end-users (Beck et al., 2001). Researchers used an Agile workflow to develop NJCoast, in which users tested the tool and provided feedback that informed modifications to the tool (Kijewski-Correa et al., 2020). Researchers developing GeoGraphVizSystem used a similar approach (Li et al., 2023). Agile research methods have been used in similar settings, such as a system for the COVID-19 pandemic response (Nazir et al., 2022). Another iterative approach employed was participatory action research (PAR), used to develop the RI-CHAMP system (Adams et al., 2024). PAR is a collaborative research approach using an iterative process encompassing a cycle of planning action, observation, and reflection, which is continued until the research problem is solved (Walker, 1993). Researchers developing RI-CHAMP solicited feedback from EM following a workshop testing the system, which was used to make improvements (Adams et al., 2024). Iterative approaches to tool development, such as Agile or PAR, support the development of tools that better reflect the needs of EM.

## Discussion

Regarding RQ1, our results suggest that there is infrequent reporting of engagement of EM in the development of DSTs for coastal storm management, as was reflected in six of the 65 articles reviewed that reported EM engagement. While EM engagement may not have been reported in the article, we feel that these results are indicative of a lack of EM engagement overall. These findings are similar to those of Newman et al. (2017), who noted that approximately 14% of the articles they reviewed mentioned EM engagement. We also found that while half of the articles reviewed made claims regarding the benefits of the tools they developed, few substantiated such claims through the successful adoption of their tool. Reporting on the outcomes of these efforts may have been omitted from the articles, or tools were implemented subsequent to the publication; however, we believe that this suggests a lack of widespread adoption and use.

Regarding RQ2, our results highlight a variety of approaches being reported by researchers to engage EM in decision support development, which we categorized into four approaches reported in the articles we reviewed. Of the six articles mentioning EM engagement, all reported engaging EM in the early stages of development. Conducting a needs assessment appeared to be an important first step in tool development, and one mentioned by all but one of the articles reviewed. Early and frequent engagement of EM has been recommended in existing DST literature (Little et al., 2015a; McIntosh et al., 2011). Training, workshops, and exercises were also commonly used approaches to test the tool in real-world conditions and solicit feedback from EM that was used to inform further design and improvement. Existing literature has noted that hands on training and exercises can ameliorate EM concerns related to the difficulties of using new tools, which may increase the likelihood adoption and use (Jennings et al., 2017). Finally, an iterative development approach was frequently reported, which has been recommended for establishing an approach to developing DSTs, specifically for aligning DSTs with changing requirements and uses over their development evolution (Gharaibeh et al., 2009).

Despite previous research identifying numerous challenges related to end-user engagement in the development and implementation of DSTs, the articles we reviewed did not report on any challenges or barriers they experienced. The omissions of challenges could reflect that none was experienced; however, we suspect that challenges were experienced but omitted in the article. Reporting on the barriers and challenges faced could potentially be useful to future efforts in developing approaches for addressing and overcoming such challenges. Of the articles we reviewed, only one mentioned successfully implementing their tool. Another mentioned that continued efforts were being made to implement the tool to support decision-making in a State Emergency Operations Center but did not report on whether they were successful in implementing the tool. Finally, one of the tools developed has been implemented for hurricane training exercises.

Our findings of approaches used to engage EM are purely a synthesis of those being reported on in existing literature, and we did not conduct further analysis to determine if certain approaches were more effective than others. Our findings provide a basic understanding of the approaches used to engage EM in the development of DSTs, which could be useful for future efforts. We believe that they highlight a disconnect between tool developers and the users of DSTs. Our results provide a preliminary understanding of the approaches currently being used to engage EM in the development of DSTs, which could be useful for future development efforts. These findings could be incorporated into future work to establish a standardized approach to developing and implementing DSTs (Walling & Vaneeckhaute, 2020). A gap in our evaluation of existing DSTs was reporting on the barriers to related to engagement and implementation, of which existing previous research has noted a variety of challenges related to co-developing and implement of DSTs (Pearman & Cravens, 2022; Stoltz et al., 2023; Walling & Vaneeckhaute, 2020). In addition, collaboration between researchers and practitioners is often rife with challenges and barriers, such as the high time commitment of collaboration, lack of funding to support collaboration, mismatch between research outputs and the needs of decision-makers, and late or insufficient engagement roadblocks to collaboration (Yates et al., 2024). A better understanding of the barrier tool developers and EM experience would be useful in informing the guidance of DST development and implementation moving forward.

In this paper, we review existing literature on coastal storm DST development, highlighting levels of engagement and approaches being used to engage EM. Overall, we found a lack of reporting in both regards. While DST development to support EM management of coastal storms is becoming increasingly prevalent in existing literature, there is an overall lack of reporting on EM engagement, as well as barriers such efforts are experiencing, which would be useful for future efforts to develop DSTs. We offer several recommendations for future work to improve DST development and implementation. First, an evaluation of existing DSTs that have achieved widespread use and adoption, such as HURREVAC, could reveal effective approaches to DST development and implementation, as well as challenges faced. Longitudinal studies or case studies assessing new DST development and implementation could be conducted to better understand the barriers and challenges, as well as approaches that are effective. Additionally, existing DST development literature has primarily focused on barriers experienced by tool developers (Pearman & Cravens, 2022; Stoltz et al., 2023). Future research could focus on those experienced by EM. Identifying such roadblocks in advance can allow for them to be addressed before considerable time, resources, and funding are invested in projects that may not succeed. For example, emergency management agencies are often financially and resource-constrained, which limits their ability to engage in activities outside of day-to-day operations (Shmueli et al., 2021), such as engaging in efforts to co-produce DSTs. In addition, it has been noted that most tools develop for EM use fail to become operational tools, and understanding the barriers could be used to develop approaches that increase their success (LaLone et al., 2023). Therefore, it would be very beneficial to understand why tools struggle to find their grounding in practice. Such an understanding could inform an approach to DST development that better addresses the needs of EMs, while researchers simultaneously focus on addressing the potential pitfalls.

## Limitations

In this section, we recognize the limitations of this research. First, we relied on two commonly used databases for conducting our review; however, other databases could have been included to increase the scope of our review and enhance our findings. However, we feel that the lack of reporting on EM engagement is an important finding in and of itself. It is also possible that researchers engaged EM in the development of DSTs, but it was not reported. Additionally, essential details regarding engagement could have been omitted from the article, either due to word limits or because the author(s) did not feel they were important. For those cases, we urge future researchers to describe these aspects of tool development in future manuscripts so that others may learn from their experience(s). Another limitation is that we did not include “Grey Literature,” or articles not published in scientific journals that could have been relevant to our review (Haddaway & Bayliss, 2015). By including Grey Literature, our review could have been more comprehensive by including government documents and reports. Government agencies are also a source of DSTs, and reviewing documents and articles in this regard could have enriched our findings. However, because our focus was on EM engagement by researchers developing DSTs, we chose to omit Grey Literature and instead concentrate on white literature (i.e., peer reviewed journal articles), where researchers tend to share their work with a goal of advancing the field. Finally, we focused on coastal storm DSTs; however, researchers in the field of natural hazards are developing DSTs for other natural hazard events, such as earthquakes, tsunamis, and wildfires, with applications for emergency management (Falter et al., 2016), as well as multi-hazard systems. A holistic review of all hazard DSTs could lead to additional insights.

## Conclusion

In this paper, we conducted a review of coastal storm DST literature to assess the engagement of EM in their development, focusing on whether EM were being engaged and the engagement approaches used, as well as outcomes of these efforts. We found limited reporting of EM engagement in the articles we reviewed, suggesting a lack of engagement overall. We also found limited reporting of successful implementation and use of DSTs by emergency managers. From the articles reporting EM engagement, we identified four approaches used to develop DSTs: (1) conduct a needs assessment to identify emergency manager needs to guide development of DST; (2) form collaborative team to engage emergency managers in tool development; (3) host workshops and training exercises with emergency mangers using the tool to solicit feedback; and (4) use an iterative design approach to incorporate emergency manager feedback into the tool. While the importance of engaging EM in the development of DSTs has been recognized, our findings suggest that there is evidence to support that EM are being engaged DST development, which may be perpetuating the lack of adoption and use. Based on our findings, we make the following recommendations: (1) evaluation and reporting on DST development and implementation in DST literature; (2) evaluation of the barriers of DST co-development and implementation, specifically those experienced by EM; and (3) the development of set of best practices or standardized guidance, such as surveying end-users before developing a tool to understand the need that informs the design and offering trainings and developing training materials, to guide future efforts by both academic researchers and government scientists to develop and implement DSTs. This review contributes to a growing body of work seeking to enhance the process for developing DSTs to ensure that they best meet the needs of end-users and are more likely to be adopted and inform decisions that potentially save lives and reduce storm damage.

## Data Availability

No datasets were generated or analysed during the current study.

## Footnotes

1 Note: it is possible that EM were engaged in the development of these tools; however, this was not mentioned in the articles reviewed.
